# Ping-Pong Mitral Stenosis: Left Atrial Myxoma With Mitral Stenosis and Pulmonary Hypertension in an Octogenarian

**DOI:** 10.14740/cr399w

**Published:** 2015-06-11

**Authors:** Prem Krishna Anandan, Natesh Bengaluru Hanumanthappa, Prabhavathi Bhat, Cholenahally Nanjappa Manjunath, Dhanalakshmi Chandrasekaran

**Affiliations:** a Sri Jayadeva Institute of Cardiovascular Research and Sciences, Bannerghatta Road, Jayanagar 9th Block, Bangalore, Karnataka 560069, India

**Keywords:** LA myxoma, Mitral stenosis, Atrial fibrillation, Pulmonary hypertension

## Abstract

Left atrial (LA) myxoma presenting with symptoms of mitral stenosis in elderly males is very rare accounting for 10% of the cases. We report an 80-year-old male who presented with symptoms of orthopnea and palpitations and was subsequently found to have a large LA myxoma obstructing the mitral valve and causing pulmonary hypertension (PHT).

## Introduction

Left atrial (LA) myxomas are the most common primary cardiac tumors and can have varied modes of presentation. Apart from embolic and systemic symptoms, they can mimic mitral stenosis when they obstruct the mitral valve. A high index of suspicion is required when they present in elderly males with symptoms of mitral valve obstruction.

## Case Report

**Figure 1:**
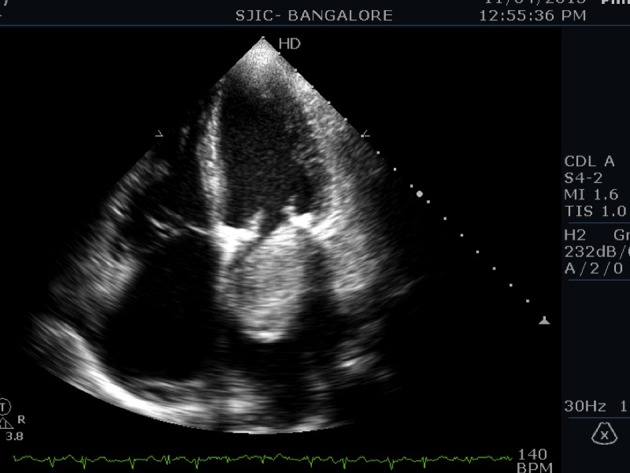
Apical four-chamber view showing a large myxoma obstructing mitral valve in diastole.

**Figure 2:**
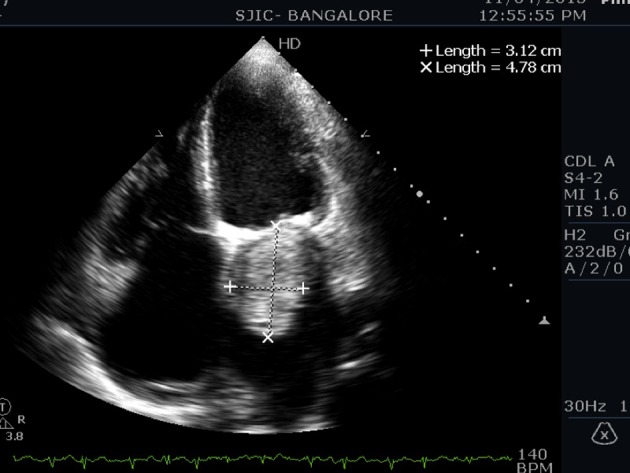
Apical four-chamber view showing a 4.7 × 3.2 cm myxoma.

**Figure 3:**
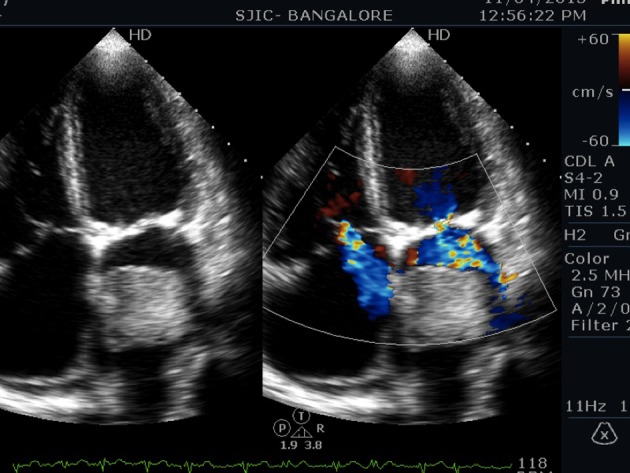
Apical four-chamber view showing moderate tricuspid regurgitation.

**Figure 4:**
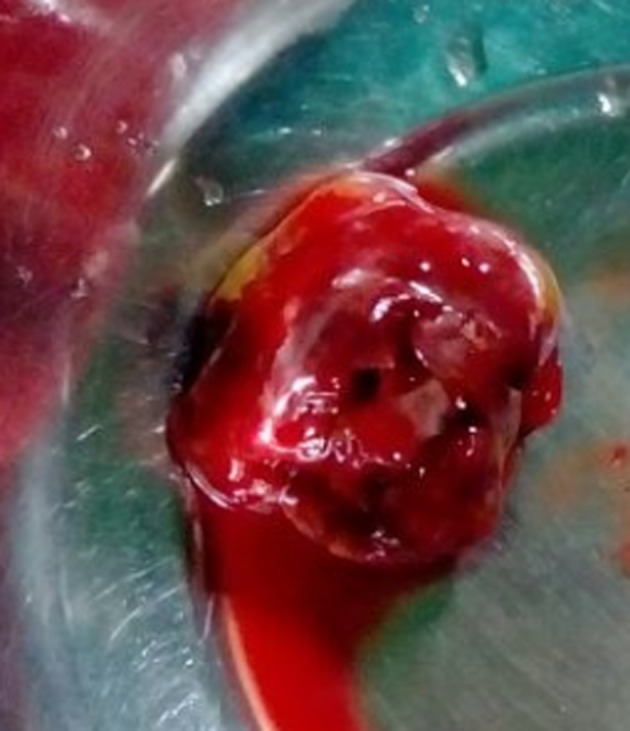
Gross specimen of surgically removed myxoma.

An 80-year-old male presented to us with symptoms of paroxysmal nocturnal dyspnea (PND), orthopnea, and palpitations for 3 months duration. Symptoms had particularly worsened after the onset of palpitations. He had no prior risk factors like diabetes, hypertension, ischemic heart disease, and hypo/hyperthyroidism. He also denied any constitutional symptoms like fever, weight loss, and arthralgia. On examination his pulse was irregular (100 beats/min), and blood pressure was 112/70 mm Hg. Cardiovascular examination revealed a variable first heart sound, mid diastolic murmur at the apex, no tumor plop, and loud pulmonary component of second heart sound. ECG showed atrial fibrillation. Chest X-ray revealed a straight left heart border with features of pulmonary venous hypertension. 2D echocardiogram (Fig. 1-3) showed a large homogenous pedunculated mass (4.7 × 3.2 cm) attached to interatrial septum obstructing the mitral valve in diastole (Supplementary video 1, www.cardiologyres.org). There was moderate tricuspid regurgitation with a TR jet of 60 mm Hg indicating pulmonary hypertension (PHT). The patient underwent successful surgical excision of the mass (Fig. 4). Histopathology was consistent with myxoma.

## Discussion

Myxoma is the most common primary cardiac tumor with an incidence of 0.0017-0.19% in autopsy series. They account for 80% of primary tumors [1]. Most common age of presentation is between 3 and 80 years with a female preponderance. Sporadic cases present in the fifth decade while familial cases present in the second decade. Familial myxomas are usually multiple with a high recurrence rate. Seventy-five percent of myxomas are located in the left atrium, 20% in the right atrium and the remaining 5% in the ventricles [2, 3]. Due to a variety of nonspecific findings at first presentation, myxoma is initially suspected in only 5% of patients [4]. Clinical manifestations can be classified into cardiac (67%), embolic (29%), and systemic (34%) symptoms [5]. Most myxomas produce symptoms when they weigh greater than 70 g. Elderly patients often present with nonspecific symptoms like fatigue, palpitations, TIA, arthralgia, and low grade fever that are often overlooked in the absence of a supporting cardiac history which makes an early diagnosis challenging. Reports similar to our case mimicking mitral stenosis, presenting with atrial fibrillation and signs of PHT have been described by Leo et al [6]. Such rare presentations account for only 10% of cases [7]. The severity of obstruction is determined by the size, location and mobility of the myxoma [8].

### Conclusion

A high index of suspicion is required to diagnose LA myxoma especially in older males where nonspecific symptoms can delay diagnosis and can adversely affect prognosis especially when atrial fibrillation and PHT have already set in.

## References

[1] Blondeau P (1990). Primary cardiac tumors--French studies of 533 cases. Thorac Cardiovasc Surg.

[2] Vale Mde P, Freire Sobrinho A, Sales MV, Teixeira MM, Cabral KC (2008). Giant myxoma in the left atrium: case report. Rev Bras Cir Cardiovasc.

[3] Diaz A, Di Salvo C, Lawrence D, Hayward M (2011). Left atrial and right ventricular myxoma: an uncommon presentation of a rare tumour. Interact Cardiovasc Thorac Surg.

[4] Goswami KC, Shrivastava S, Bahl VK, Saxena A, Manchanda SC, Wasir HS (1998). Cardiac myxomas: clinical and echocardiographic profile. Int J Cardiol.

[5] Pinede L, Duhaut P, Loire R (2001). Clinical presentation of left atrial cardiac myxoma. A series of 112 consecutive cases. Medicine (Baltimore).

[6] Leo S, Yang K, Weng C, Liang Z (2013). Large atrial myxoma mimicking severe mitral stenosis associated with right heart enlargement and severe pulmonary hypertension. Cardiovasc Diagn Ther.

[7] Ojji DB, Mamven MH, Omonua O, Habib Z, Osaze H, Sliwa K (2012). Left atrial myxoma mimicking mitral stenosis. Clin Med Insights Case Rep.

[8] Carney JA (1995). The Carney complex (myxomas, spotty pigmentation, endocrine overactivity, and schwannomas). Dermatol Clin.

